# Haloamines of the Neurotransmitter γ-Aminobutyric Acid (GABA) and Its Ethyl Ester: Mild Oxidants for Reactions in Hydrophobic Microenvironments and Bactericidal Activity

**DOI:** 10.3390/molecules30214227

**Published:** 2025-10-29

**Authors:** Luiza de Carvalho Bertozo, Markus Nagl, Valdecir Farias Ximenes

**Affiliations:** 1 São Paulo State University (UNESP), School of Sciences, Bauru 17033-360, Brazil; luiza.bertozo@unesp.br; 2 Institute of Hygiene and Medical Microbiology, Medical University of Innsbruck, 6020 Innsbruck, Austria; m.nagl@i-med.ac.at

**Keywords:** GABA, taurine, chloramines, bromamines, micelles, bactericidal activity, antiseptic

## Abstract

N-chlorotaurine (Tau-Cl) is a mild oxidizing haloamine formed from the reaction of hypochlorous acid (HOCl) with taurine (2-amino-ethanesulfonic acid). It is widely used as a topical antiseptic. In this study, we investigated haloamines derived from the neurotransmitter γ-aminobutyric acid, specifically GABA chloramine and bromamine (GABA-Cl, GABA-Br), as well as their halogenated γ-aminobutyric acid ethyl esters (GABAet-Cl, GABAet-Br). Due to their higher hydrophobicity, the esterified haloamines were more potent oxidants in the presence of lyophilic surfactant micelles, demonstrating their greater ability to access hydrophobic environments. By using fluorescent azapentalenes as molecular targets incorporated into sodium dodecyl sulfate (SDS) micelles, the second-order oxidation rate constants (k_2_) resulted in 1.15 × 10^2^ and 1.10 × 10^4^ M^−1^min^−1^ for GABA-Cl and GABAet-Cl, respectively. As expected, due to the presence of a bromine atom, GABAet-Br was even more reactive (4.50 × 10^6^ M^−1^min^−1^). The ability of GABAet-Br to access hydrophobic sites was demonstrated by comparing the reaction rate using micelles generated by different surfactants such as SDS (4.5 × 10^6^ M^−1^min^−1^), cetyltrimethylammonium chloride (CTAC, 2.5 × 10^4^ M^−1^min^−1^), and triton X-100 (TX-100, 3.9 × 10^3^ M^−1^min^−1^). GABAet-Cl and GABAet-Br exhibited higher bactericidal activity against *Staphylococcus aureus* and *Escherichia coli*, probably due to their increased lipophilicity and improved penetration into microorganisms compared to GABA-Cl and GABA-Br. The enhancement of the oxidation capacity by GABAet-Cl and GABAet-Br represents a new direction in the exploration and application of haloamines as antiseptic agents.

## 1. Introduction

Chloramines are the products of the reaction between hypochlorous acid (HOCl) and amines. In the innate immune system, chloramines gained relevance in the 1980s when taurine (2-aminoethanesulfonic acid) was used to trap HOCl generated by stimulated human neutrophils, and its product, N-chlorotaurine (taurine chloramine, Tau-Cl), was identified [1]. Taurine is a non-essential amino acid abundant in all mammalian tissues. Taurine is particularly notable among the endogenous amine substrates that react with HOCl produced by neutrophils, as intracellular taurine levels reach around 20 mM. Together with other amines, taurine makes up 90% of the N-chloramine derivatives that accumulate in stimulated neutrophils [2,3]. Activated neutrophils produce high amounts of HOCl by oxidizing chloride with hydrogen peroxide (H_2_O_2_), catalyzed by myeloperoxidase (MPO). The involvement of MPO and HOCl in the pathogenesis of inflammatory diseases is widely accepted [4,5,6,7].

Tau-Cl is a long-lasting and mild oxidant compared to the precursor HOCl [8]. This chemical property is central to its broad biological application as an anti-infective and anti-inflammatory compound [8,9,10,11]. The potential therapeutic application of Tau-Cl has been shown in clinical trials. For instance, a Phase-1 clinical trial was conducted to study the tolerability of inhaled 1% Tau-Cl in an aqueous solution upon repeated application [12]. A Phase 2 clinical trial demonstrated the tolerability and efficacy of Tau-Cl in epidemic keratoconjunctivitis [13].

N-bromotaurine (Taurine bromamine (Tau-Br) is the bromine equivalent of Tau-Cl. Tau-Br is produced by the reaction between hypobromous acid (HOBr) and taurine and, similarly to Tau-Cl, it is a natural oxidizing taurine derivative produced by inflammatory cells [14]. At inflammatory sites, HOBr is generated through the oxidation of bromide by H_2_O_2_ in a reaction catalyzed by MPO or eosinophil peroxidase (EPO) [15]. The main difference between HOCl and HOBr is the higher reactivity of the latter. For instance, HOBr promotes the halogenation of tyrosine approximately 5000-fold faster than HOCl [16]. This chemical feature of HOBr, which is related to the higher electrophilic character of bromine, is extended to Tau-Br, which is also significantly more reactive than Tau-Cl [17,18,19]. As expected, Tau-Br and related compounds, such as bromamine T, also exhibit microbicidal and anti-inflammatory properties and have been widely studied [14,20,21,22].

Besides its mild oxidizing character, the relative chemical stability of Tau-Cl explains its broad application as a microbicidal agent. The stability of Tau-Cl relies on its β-amino acid structure. In contrast, the chloramines from α-amino acids are unstable and decompose to the corresponding aldehydes. The instability is due to the carboxylic group’s proximity to the chloramine unit, which favors the formation of an imine intermediate, followed by hydrolysis into aldehydes [23]. Conversely, Tau-Cl, as a β-amino acid, is much more stable, and one factor contributing to its higher stability is that the chloramine group is two carbon atoms away from the sulfonyl group [23,24]. Based on this chemical feature of haloamines derived from amino acids, the present work was conceived, namely to study of the chemical properties of haloamines derived from the neurotransmitter γ-aminobutyric acid (GABA). Since the amino group is three carbon atoms away from the carboxyl group, the generated chloramine would be as stable as Tau-Cl, which was indeed confirmed. Another motivating factor for this work was the recent discovery that the ester derivative of the GABA exhibited significantly higher reactivity in a micellar environment [25]. This finding is highly relevant, as access to cell membranes can improve their microbicidal properties compared to Tau-Cl.

Considering the above, we aimed to evaluate the reactivity of GABA haloamines and their esters using several targets incorporated into micelles, which could simulate the effectiveness of these compounds in accessing cell membranes. This experimental model successfully demonstrated the importance of the hydrophobic nature of these oxidants in accessing a hydrophobic microenvironment. Supporting our hypothesis, we also demonstrated the efficacy of these new compounds as bactericidal agents.

## 2. Results and Discussion

### 2.1. Preparation and Stability of Solutions of Haloamines

**Figure 1:**
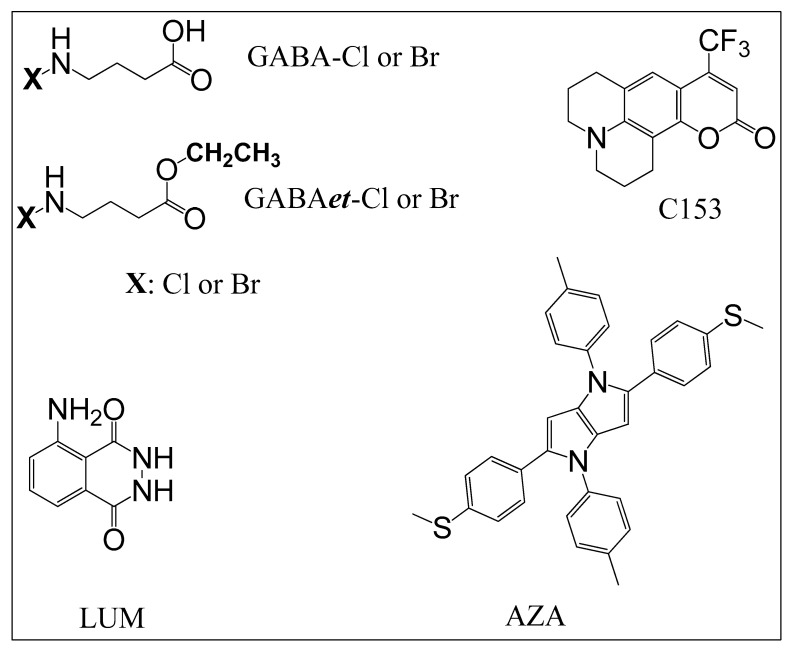
Molecular structures of haloamines and oxidizable molecular targets. Chloramine or bromamine of GABA (GABA-Cl, GABA-Br), chloramine or bromamine of the ethyl ester of GABA (GABAet-Cl, GABAet-Br), coumarin 153 (C153), azapentalene (AZA), and luminol (LUM).

This work was conceived based on the broad biomedical applications of Tau-Cl, a microbicidal agent [9,11,26,27]. To further our understanding and applications of Tau-Cl and related compounds, we focused on exploring the chemical properties of haloamine derivatives of GABA and its ethyl ester, GABAet. The molecular structure of the haloamines and the molecular targets used to evaluate their reactivity are depicted in Figure 1.

Solutions of Tau-Cl can be easily prepared by mixing HOCl with an excess of taurine [27,28,29,30]. We used this protocol to prepare solutions of GABA chloramine (GABA-Cl) and its ethyl ester derivative (GABAet-Cl). Specifically, the chloramine solutions were prepared by combining HOCl with a 10-fold excess of GABA or GABAet in PBS at pH 7.4. Figure S1A shows the UV-Vis spectra of Tau-Cl, GABA-Cl, and GABAet-Cl, which are consistent with the reported spectrum that peaks at 250 nm. As expected, the overlapping spectra of Tau-Cl, GABA-Cl, and GABAet-Cl evidenced the formation of the chloramines. Similarly, Figure S1B shows the overlay of the UV-Vis spectra of Tau-Br, GABA-Br, and GABAet-Br with their characteristic band peaking at 288 nm [28,31].

**Figure 2:**
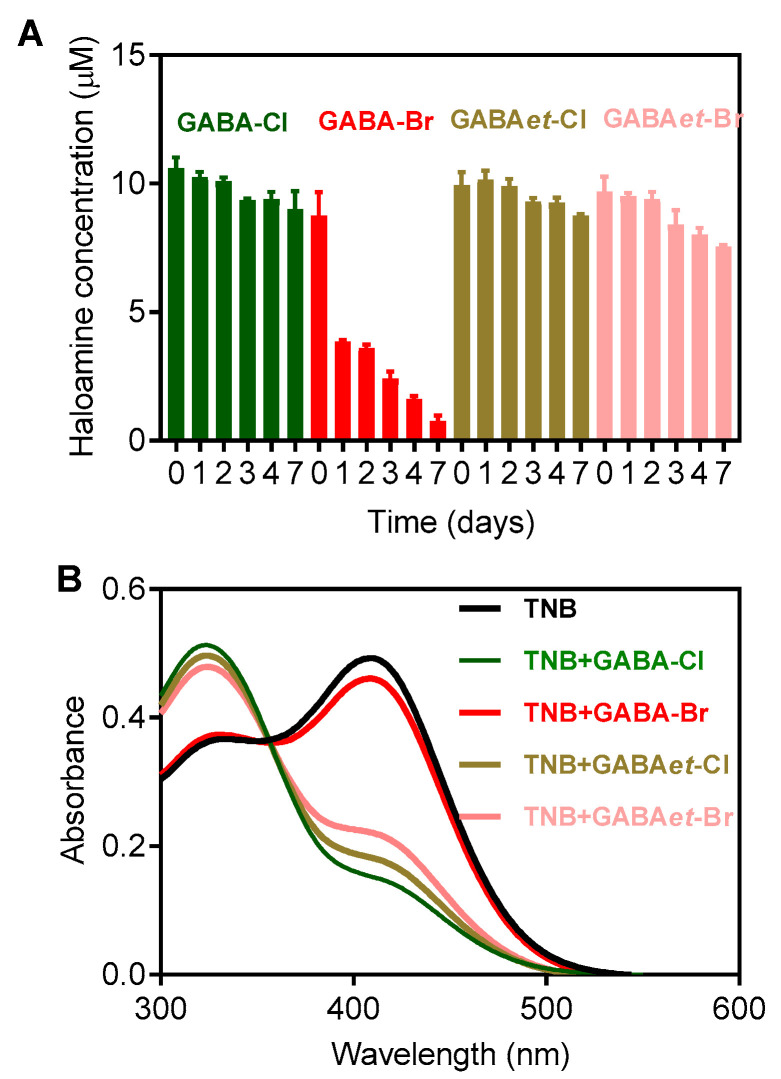
Study on the stability of haloamines. Haloamines solutions were prepared as described in Section 3 and analyzed for their capacity to oxidize TNB. (**A**) Concentrations of haloamines were measured one hour after preparation and over a period of 7 days. (**B**) TNB spectra recorded before and after the addition of haloamines. The spectra show the measurement taken on the seventh day. The solutions were kept at 4 °C. The results are shown as mean values ± SD of three independent experiments.

The stability of the haloamines was measured by monitoring the conservation of their oxidizing power using TNB as a reducing agent [23]. Figure 2 illustrates the degradation of the haloamines over a seven-day period for solutions stored in the refrigerator. The solutions of GABA-Cl and GABAet-Cl were fairly stable, which is consistent with the literature data for Tau-Cl. GABA-Br was less stable than GABA-Cl, which follows the behavior of Tau-Br compared to Tau-Cl [20]. However, an important finding was the observation that GABAet-Br was significantly more stable than GABA-Br, which could be an additional advantage for its application as a microbicidal agent. Upon analyzing the molecular structure of GABA-Cl and the ester derivative GABAet-Cl, it is evident that the chloramine reactive moiety (N-Cl) remains unchanged (Figure 1). Therefore, the findings outlined in the manuscript demonstrate that the increased reactivity of GABAet-Cl compared to GABA-Cl, as well as GABA-Br compared to GABAet-Br, is attributed to their hydrophobic nature, which, in turn, enables better access to hydrophobic environments.

### 2.2. The Impact of Esterification on the Reactivity of GABA Chloramine in SDS Micelles

Chloramines act as mild oxidizing agents, promoting the selective oxidation of sulfhydryl and methionine residues in proteins [32,33,34,35]. Based on this property, and to compare the reactivity of GABA-Cl and GABAet-Cl, a fluorescent azapentalene bearing a methyl sulfide substituent was chosen as a target (AZA, Figure 1). The reaction of AZA with HOCl resulted in the oxidation of the sulfhydryl group to sulfoxide and the subsequent loss of fluorescence [25].

**Figure 3:**
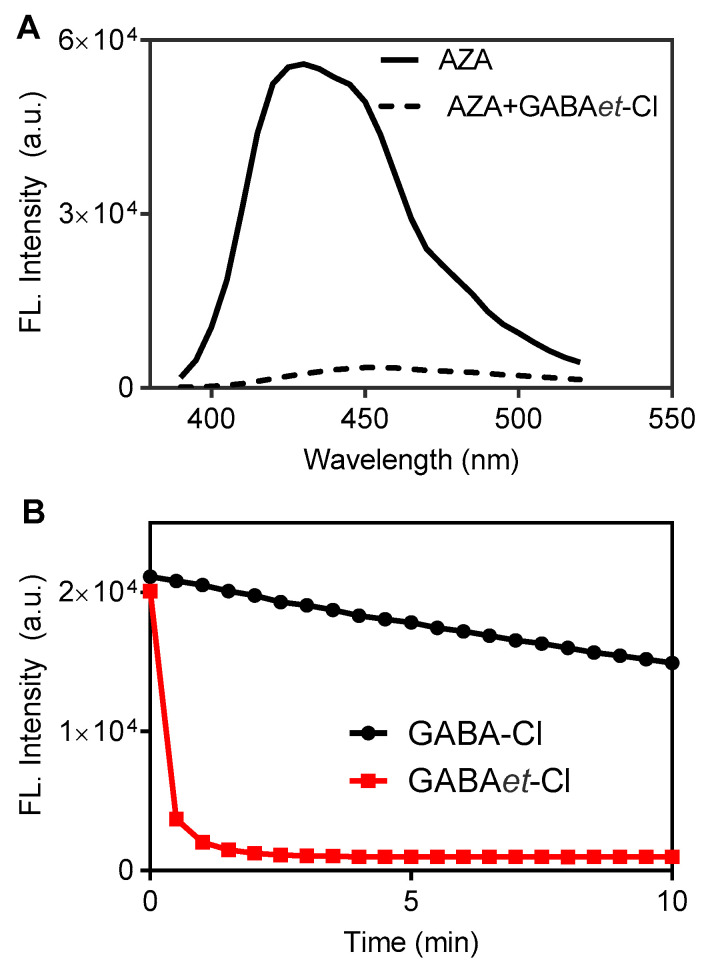
Oxidation of AZA: GABA-Cl versus GABAet-Cl in SDS micelles. (**A**) AZA emission spectrum before and after the addition of GABAet-Cl. (**B**) Time-dependent emission decay (360/430 nm). Final concentrations: SDS 40 mM in PBS pH 7.4, AZA 10 μM, GABA-Cl or GABAet-Cl 200 μM.

As will be shown in the following sections, the core of the present work was to demonstrate that the haloamines of GABA can access hydrophobic microenvironments and increase their reactivity with the targets. Here, the surfactants used were the classic representatives of neutral (TX-100), positively charged (CTAC), and negatively charged (SDS) surfactants. The micelles of SDS were previously prepared using a surfactant concentration 5-fold higher than the critical micellar concentration. Then, AZA was incorporated into micelles before the oxidants were added. As a highly hydrophobic compound, the incorporation of AZA was previously demonstrated in a paper by our group and was used to determine the critical micelle concentration (CMC) of SDS [25]. As expected, GABA-Cl and GABAet-Cl oxidized AZA in the micellar medium. However, GABAet-Cl was significantly more reactive. Figure 3A shows AZA’s fluorescence spectrum before and after the addition of GABAet-Cl, and Figure 3B compares the fluorescence decay caused by GABA-Cl and GABAet-Cl. In these experiments, micelles of SDS were previously prepared using a surfactant concentration 5 times higher than the critical micellar concentration. The finding in Figure 3B suggests that GABA-Cl and GABAet-Cl must diffuse to the micelle core to oxidize AZA. Thus, considering that no difference between GABA-Cl and GABAet-Cl was detected in the absence of SDS, i.e., there was no difference in the reaction in the bulk solution (Figure S2), these findings must be related to the higher accessibility of GABAet-Cl to the micelle core.

**Figure 4:**
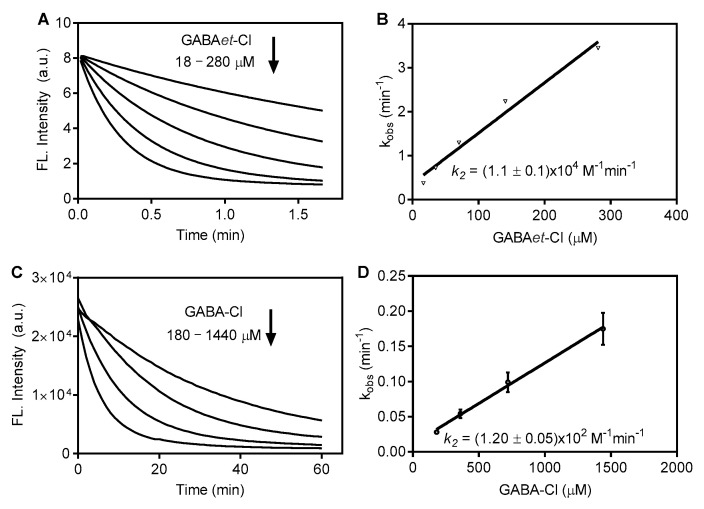
Oxidation of AZA in SDS micelles: GABA-Cl versus GABAet-Cl. Time-dependent fluorescence decay and determination of the second-order rate constant (k_2_) for the reaction with (**A**,**B**) GABAet-Cl and (**C**,**D**) GABA-Cl. Final concentrations: SDS 40 mM in PBS pH 7.4, AZA 6.25 μM, GABA-Cl or GABAet-Cl as indicated. The results are shown as mean values ± SD of three independent experiments.

To gain a deep comprehension of the difference in the reactivity of GABA-Cl and GABAet-Cl, AZA’s fluorescence decay was measured by stopped-flow (Figure 4). In these experiments, the concentration of AZA was kept constant at 6.5 μM, and GABA-Cl was varied from 180 to 1440 μM. Under this pseudo-first-order experimental condition, the observed first-order constants (k_obs_) were measured, and the second-order rate constant (k_2_) was calculated, resulting in 115 M^−1^min^−1^. For GABAet-Cl, which was more reactive, the concentration varied from 18 to 180 μM, and k_2_ was found to be 1.1 × 10^4^ M^−1^min^−1^. In short, the esterification leads to a two-order-of-magnitude increase in the rate constant. Since the reactive chloramine group is not altered in both compounds, this effect can be attributed to the higher accessibility of the ester derivative to the micelle core.

**Figure 5:**
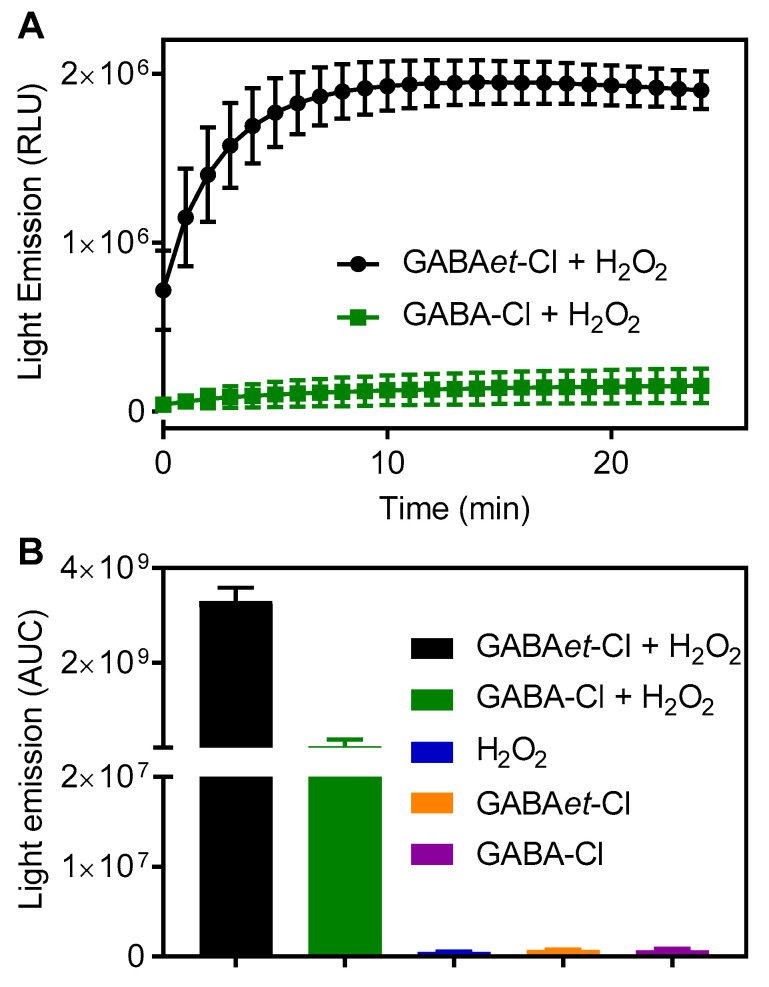
Light emission from luminol oxidation: GABAet-Cl versus GABA-Cl. (**A**) Kinetic profile of the reaction. (**B**) Integrated light emission. Final concentrations: SDS 40 mM in PBS pH 7.4, GABAet-Cl or GABA-Cl 100 μM, H_2_O_2_ 100 μM, luminol 25 μM. The results are shown as mean values ± SD of three independent experiments.

Luminol is a chemiluminescent probe, and light emission can be enhanced in micellar media [36,37]. In addition, Tau-Cl associated with H_2_O_2_ has been identified as an efficient oxidant of luminol oxidation, probably through the generation of singlet oxygen. Based on the literature data, the oxidative potency of GABA-Cl and GABAet-Cl associated with H_2_O_2_ was also compared using luminol in SDS. The reaction efficiency was monitored by light emission. The results shown in Figure 5 confirmed the previous findings. As depicted, the chemiluminescence was dependent on both chloramines and H_2_O_2_. Furthermore, a significant difference was observed, with GABAet-Cl being the most effective oxidant in the micellar medium. As a control, H_2_O_2_, GABA-Cl, and GABAet-Cl alone were ineffective in the micellar media. Based on these findings, the greater accessibility of GABAet-Cl to the micelle core could once again account for these results.

### 2.3. Increasing Reactivity: GABAet-Cl Versus GABAet-Br in SDS Micelles

Due to the higher electrophilic strength of the intermediate species bromonium (Br^+^) versus chloronium (Cl^+^) ions, HOBr is well-known for its increased reactivity compared to HOCl [38,39,40,41,42,43,44,45,46,47,48,49,50]. This chemical property extends to its haloamine derivatives. Tau-Br can oxidize several biomolecules that are insensitive to Tau-Cl [17,19,21]. Hence, the next task was to compare GABAet-Cl with GABAet-Br. It must be emphasized that both were prepared in the same way, the only difference being the substitution of HOCl by HOBr.

To compare the reactivity of GABAet-Cl with GABAet-Br, the oxidizable probe was replaced with coumarin 153 (C153). This was done because C153 has lower reactivity, as it lacks AZA’s methyl-sulfide moiety, making it easier to detect differences in reactivity between GABAet-Cl and GABAet-Br. It is worth mentioning that C153 is a fluorescent compound widely used to determine the critical micelle concentration (CMC) of micelles, indicating its affinity for hydrophobic cores [51].

The results in Figure S3 confirmed our expectations, showing that GABAet-Br, but not GABAet-Cl, oxidized C153. This result is consistent with the well-established higher reactivity of bromine derivatives [17,19,21,38,39]. To evaluate the effect of the micellar system, GABAet-Br and GABA-Br were compared, with C153 as the target.

**Figure 6:**
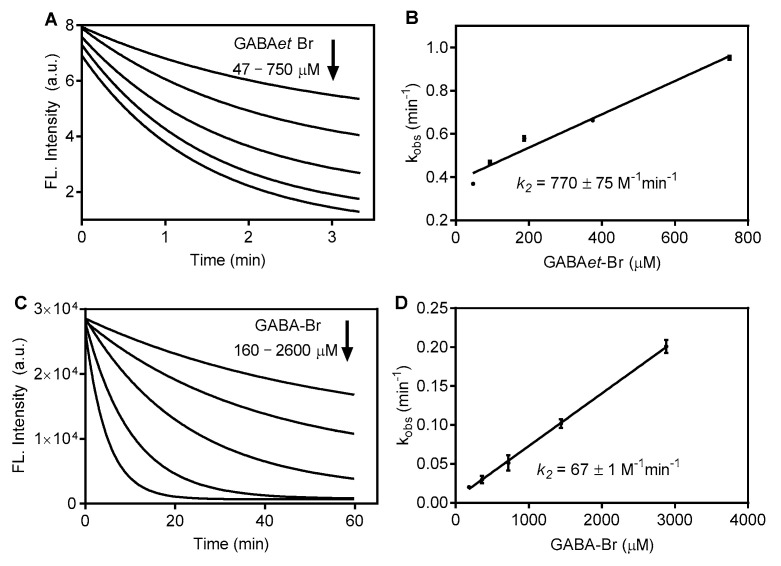
Oxidation of C153 in SDS micelles: GABAet-Br versus GABA-Br. Time-dependent fluorescence decay and determination of the second-order rate constant (k_2_) for the reaction with (**A**,**B**) GABAet-Br and (**C**,**D**) GABA-Br. Final concentrations: SDS 40 mM in PBS pH 7.4, C153 12.5 μM, GABA-Br or GABAet-Br as indicated. The results are shown as mean values ± SD of three independent experiments.

The results in Figure 6A,B show the fluorescence decay of C153 caused by GABAet-Br and GABA-Br. The second-order rate constants were 770 M^−1^min^−1^ and 67 M^−1^min^−1^ for GABAet-Br and GABA-Br, respectively, confirming the previous results and highlighting the relevance of the hydrophobicity as a determinant for accessing the micelle core. It is worth noting the significantly lower rate constants for the oxidation of C153 compared to AZA, which is attributed to the lower oxidizability of the coumarin. This chemical feature also explains the unreactivity of C153 with GABAet-Cl (Figure S3).

### 2.4. The Nature of the Surfactants

To emphasize the importance of evaluating the micelle core, additional surfactants were examined. The idea was to compare the efficiency of oxidation in SDS (negatively charged micelles) with CTAC (positively charged micelles) and TX-100 (neutral micelles). Besides the surface charge, these surfactants produce micelles of different radii, a feature that must be considered when assessing the haloamines with respect to the targets. In this regard, the radii and shape of micelles depend not only on their chemical nature, but also on the concentration of the surfactant in solution. The typical values are 17–18 Å for SDS [41], 20–30 Å for CTAC [42], and ∼40 Å for TX-100 [43]. Here, the micelles were prepared by dissolving the surfactants at a concentration at least five times higher than their critical micelle concentrations (CMCs) [52]. It is also important to note that the choice of CTAC instead of the most commonly used surfactant cetyltrimethylammonium bromide (CTAB) was made to avoid the presence of the counterion bromide in the medium, which could bias the comparison between GABAet-Cl and GABAet-Br, since the reaction between GABAet-Cl and Br can form the latter, as shown below.

The effect of the chemical nature of the surfactant was promptly evidenced. Notably, the second-order rate constant for the reaction of GABAet-Cl with AZA was 1.1 × 10^4^ M^−1^min^−1^ in SDS (Figure 4), but it was not reactive in CTAC or TX-100. However, by adding bromide ions (Br^−^) to the reaction medium, the reactivity was increased (Figure S4). The explanation here relies on the in situ formation of GABAet-Br or free bromine through the reaction between GABAet-Cl and Br^−^. Overall, these results are consistent with the well-established higher reactivity of Tau-Br compared to Tau-Cl [17,18,19].

**Figure 7:**
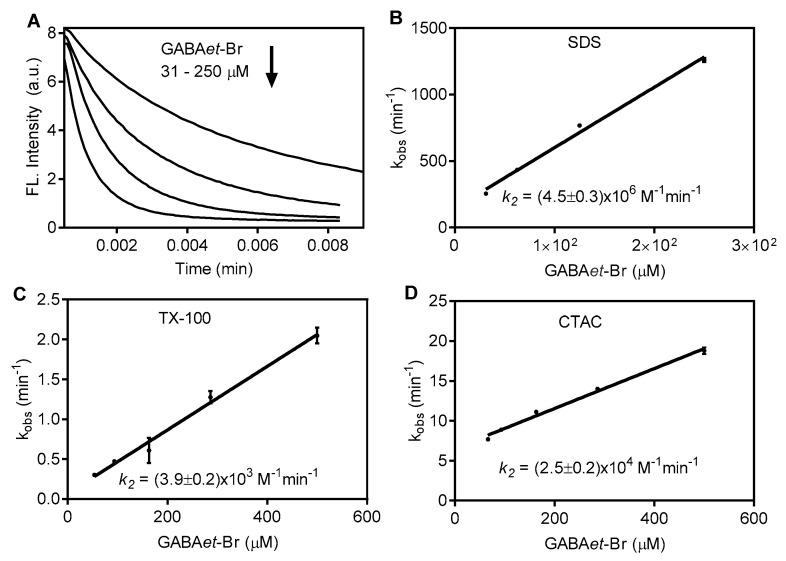
Effect of surfactants on AZA oxidation by GABAet-Br. (**A**) Fluorescence decay in SDS. (**B**–**D**) Determination of the second-order rate constants (k_2_). Final concentrations: SDS 40 mM, CTAC 5 mM, TX-100 1.25 mM in PBS pH 7.4, AZA 6.25 μM, and GABAet-Br as indicated. The results are shown as mean values ± SD of three independent experiments.

There are two possible explanations for the lower reactivity in CTAC and TX-100. The first one is related to the micelle surface charge. However, there is no reason to suspect that a neutral molecule (AZA) would prefer a negative micelle over a positive or neutral one. Corroborating this, the higher reactivity of GABAet-Br compared to GABA-Br was also verified in neutral TX-100 micelles, indicating that a negative charge on the surface of the micelle is not a prerequisite for the reaction (Figure S5). The second and more plausible explanation relates to the micelle size. It can be inferred that access to GABAet-Cl would be more difficult in TX-100 and CTAC compared to the smaller SDS micelles. In the same direction, the reactivity of GABAet-Br with AZA decreased in CTAC and TX-100 compared to SDS. Figure 7 shows the second-order rate for the reaction in SDS (4.5 × 10^6^ M^−1^min^−1^), CTAC (2.5 × 10^4^ M^−1^min^−1^), and TX-100 (3.9 × 10^3^ M^−1^min^−1^). These results confirmed the necessity of entering micelles and the relevance of the hydrophobic nature of haloamines.

### 2.5. Competitive Inhibition of C153 Oxidation

**Figure 8:**
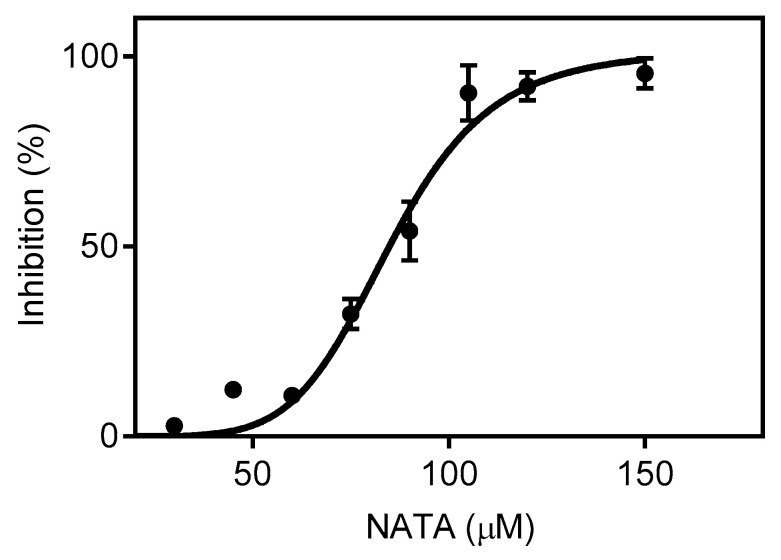
Inhibitory effect of NATA on C153 oxidation by GABAet-Br. Experimental conditions: SDS 40 mM, C153 12.5 μM in PBS, pH 7.4, GABAet-Br 200 μM, and NATA as indicated. The results are shown as mean values ± SD of three independent experiments.

The finding that GABA ester haloamines can access the micelle core is supported by a competitive experiment in which NATA, a lipophilic derivative of tryptophan, was previously incorporated into the micelle. The choice of NATA was based on the well-known reactivity of tryptophan and its derivatives with bromamines [17] and the known incorporation of NATA in micelles [44]. Figure 8 shows the inhibitory effect due to increasing concentrations of NATA on the oxidation of C153 by GABAet-Br. Considering that C153 was used at a concentration of 12.5 μM and significant inhibition was only observed at concentrations above 50 μM, this result indicates the higher reactivity of C153 compared to NATA. Overall, these findings provide additional evidence for a reaction occurring at the micelle core.

### 2.6. Oxidation of Tryptophan Residues in Albumin

**Figure 9:**
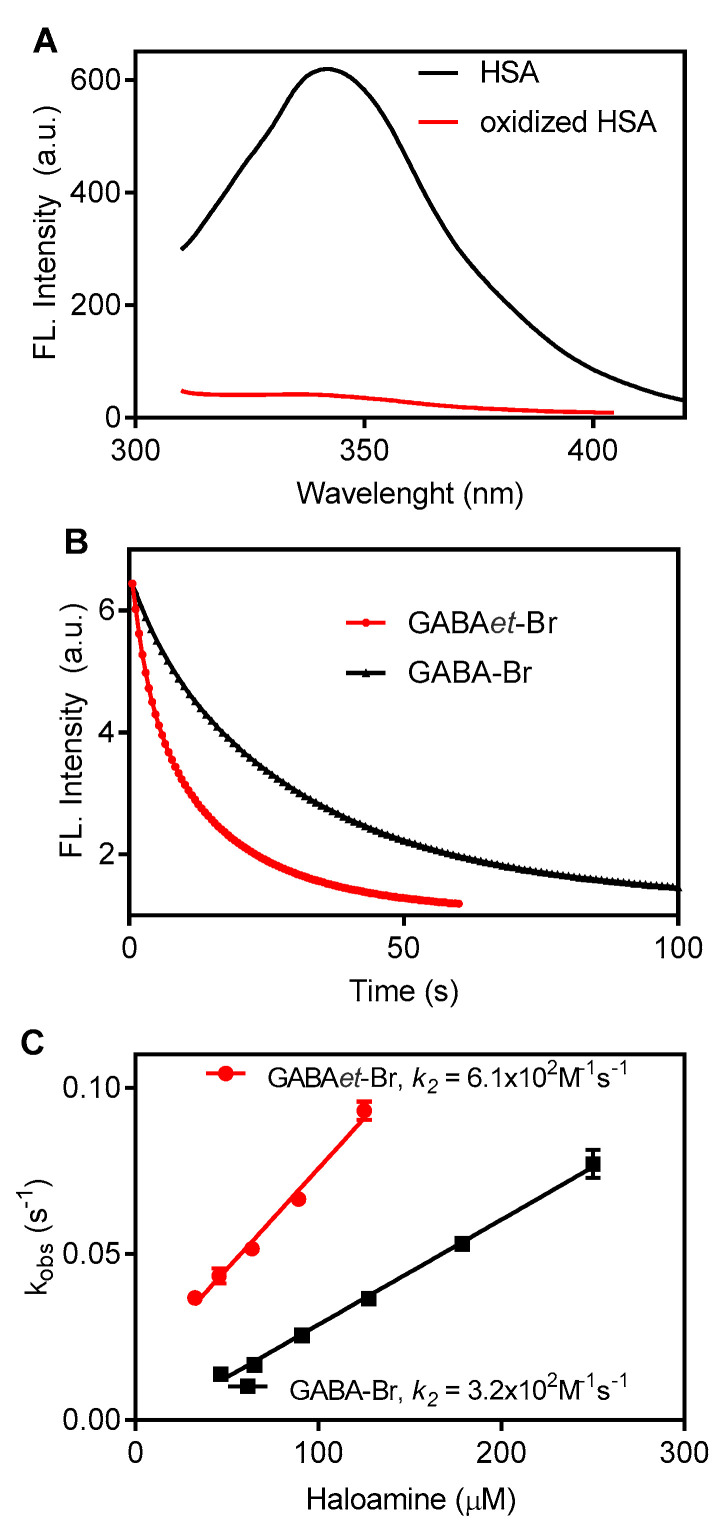
Oxidation of HSA: GABA-Br versus GABAet-Br: (**A**) Intrinsic fluorescence spectrum of native and oxidized HSA. (**B**) Time-dependent decay of intrinsic fluorescence of HSA (HSA 5 μM, haloamines 125 μM). (**C**) Determination of second-order rate constants. Reaction conditions: HSA 5.0 μM, haloamines (30–250 μM) in PBS at pH 7.4.

The higher capacity of GABAet-Br to access hydrophobic microenvironments compared to GABA-Br was also evidenced by studying the oxidation of human serum albumin (HSA). The study was based on the oxidation of the single tryptophan residue (TRP214) located at site-I of HSA, which is a hydrophobic cavity within the subdomain IIA of HSA and plays a fundamental role as a binding site for transportation of poorly water-soluble drugs in blood plasma [45,46]. Due to its hydrophobic nature, low-polarity compounds, including fatty acids, are potent ligands for HSA [47,48]. Hence, it is reasonable to expect that GABAet-Br has higher accessibility to site I than GABA-Br, enabling the oxidation of TRP214. This expectation was confirmed, as depicted in Figure 9. Specifically, Figure 9A shows that GABA-Br and GABAet-Br were able to deplete HSA’s intrinsic fluorescence, providing evidence of TRP214 oxidation, which has been demonstrated using HOCl, HOBr, and Tau-Br [17,49,50]. Figure 9B shows the time-dependent depletion of TRP214, and Figure 9C presents the second-order rate constants. Even though the difference was smaller than that observed in micelle systems, a two-fold higher efficiency was detected for GABAet-Br compared to GABA-Br.

### 2.7. Application as Bactericidal Agents

Considering the higher oxidation power of the ester derivatives and their relationship with the enhanced capacity to access the hydrophobic microenvironment, it can be inferred that these compounds may also be more effective microbicidal agents. Thus, to validate this hypothesis, the bactericidal efficacy of GABA-Cl, GABAet-Cl, GABA-Br, and GABAet-Br was evaluated against *S. aureus* and *E. coli*.

**Figure 10:**
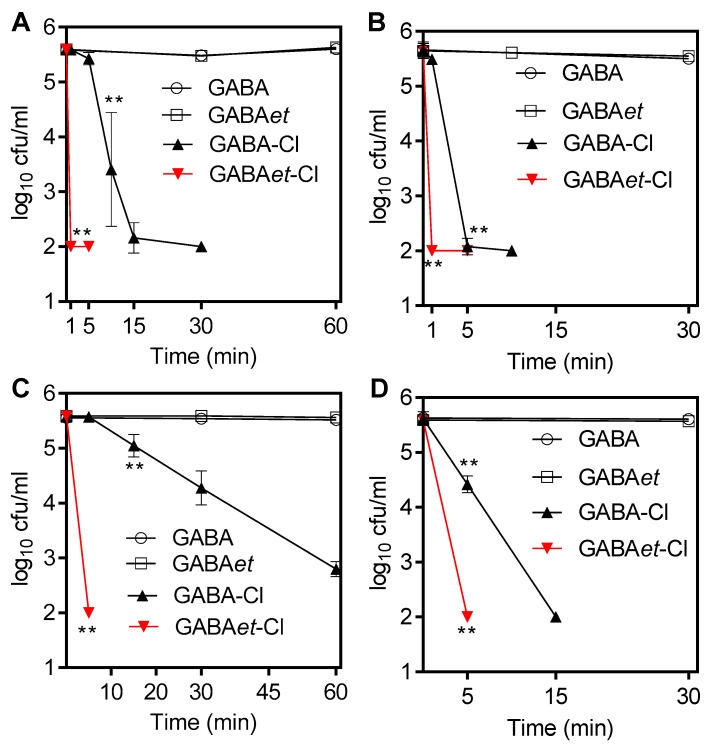
Activity of GABA-Cl and GABAet-Cl at 50 mM (**A**,**B**) and 10 mM (**C**,**D**) against *S. aureus* (**A**,**C**) and *E. coli* (**B**,**D**). Mean values ± SD of three independent experiments. ** *p* < 0.01 versus controls and between GABA-Cl and GABAet-Cl. Thresholds of significance are shown.

Corroborating our expectations, GABA-Cl at a concentration of 50 mM killed *S. aureus* nearly to the detection limit after 15 min and *E. coli* after only 5 min at 37 °C. GABAet-Cl had an even more potent activity and completely killed both test strains within 1 min at a concentration of 50 mM (Figure 10A,B). The killing by 10 mM GABA-Cl was slower, with 60 min needed to approach the detection limit of *S. aureus*, which was reached after 15 min for *E. coli*. In contrast, 5 min at 10 mM was sufficient for GABAet-Cl (Figure 10C,D). As a control, the precursor compounds GABA and GABAet (100 mM) had no bactericidal activity against *S. aureus* and *E. coli*.

**Figure 11:**
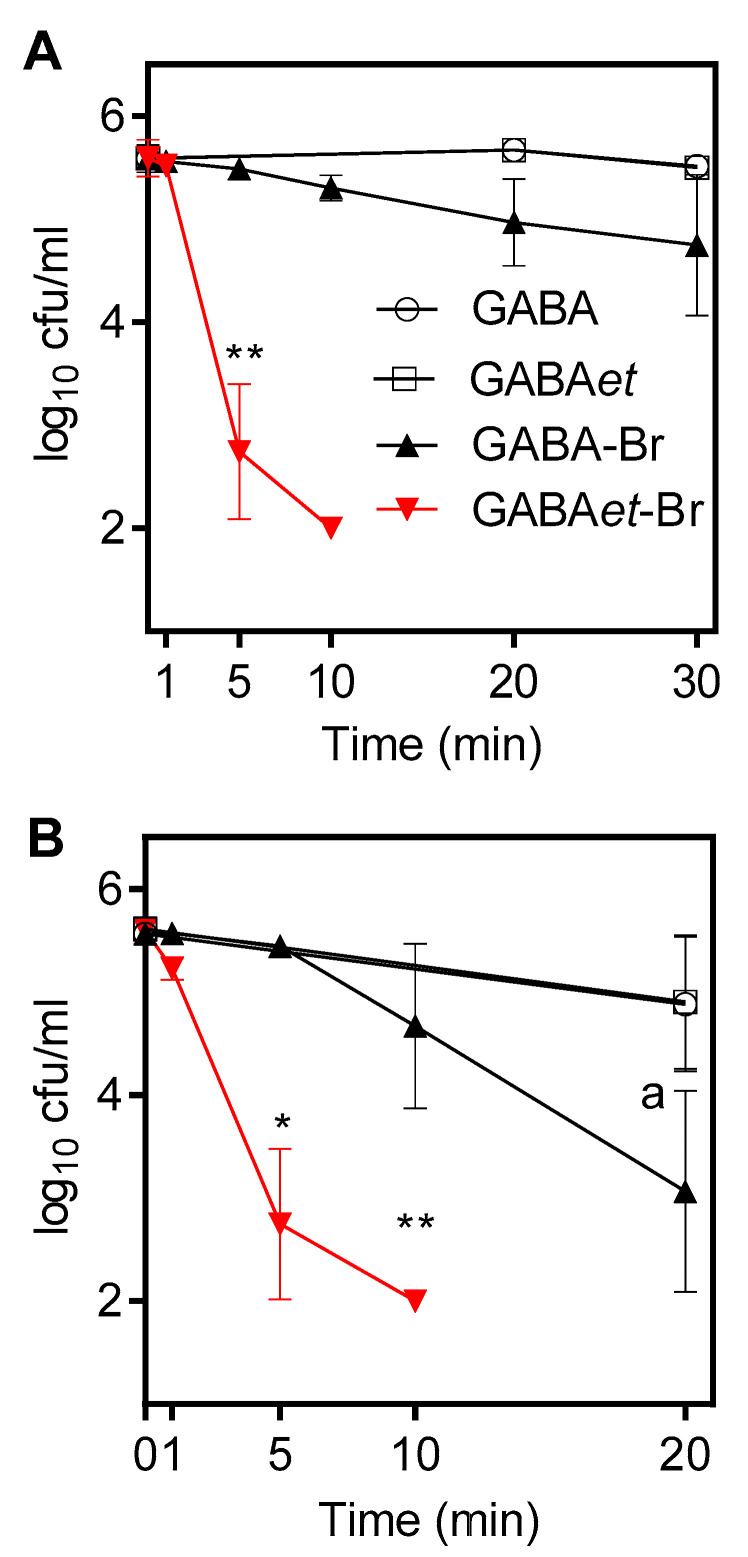
Activity of GABA-Br and GABAet-Br at 10 microM against (**A**) *S. aureus* and (**B**) *E. coli*. Mean values ± SD of three independent experiments. * *p* < 0.05 and ** *p* < 0.01 versus controls and between GABA-Br and GABAet-Br; ^a^ *p* = 0.056 versus control (GABA).

Bromamines generally exhibit significantly more potent bactericidal activity in plain buffer solutions than chloramines; therefore, we applied a typical micromolar concentration of these compounds. Consistent with this expectation, GABA-Br and GABAet-Br demonstrated markedly higher bactericidal activity, typical of active bromine compounds [10,19,22,53]. GABA-Br and GABAet-Br at a 1000-fold lower concentration (10 µM) killed both test strains (Figure 11A,B). Again, GABAet-Br, with killing times of 5 min for *E. coli* and 10 min for *S. aureus* to reach the detection limit, exhibited more potent bactericidal activity than GABA-Br, with respective times of >20 min for *E. coli* and *S. aureus*. Finally, *E. coli*, as a Gram-negative bacterium, was slightly more susceptible than *S. aureus*, a Gram-positive bacterium, which is typical of active halogen compounds [10]. The more pronounced bactericidal activity of the esterified haloamines is likely due to their higher lipophilicity and improved penetration into microorganisms.

## 3. Materials and Methods

### 3.1. Chemicals

Gamma-aminobutyric acid (GABA), ethyl 4-aminobutyrate hydrochloride (GABAet), N-acetyl-L-tryptophanamide (NATA), sodium dodecyl sulfate (SDS), cetyltrimethylammonium chloride (CTAC), Triton X-100 (TX-100), coumarin 153 (C153), human serum albumin (HSA) free of fatty acid and globulins (A3782), luminol, 2-aminoethanesulfonic acid (taurine), sodium hypochlorite solution (12–15% free chlorine), 5,5′-dithio-bis-(2-nitrobenzoic acid) (DTNB) and hydrogen peroxide (H_2_O_2_) (30%) were purchased from Sigma-Aldrich Chemical Co. (St. Louis, MO, USA). 2,5-Bis-(4-methylsulfanyl-phenyl)-1,4-di-p-tolyl-1,4-dihydro-pyrrolo[3,2-b]pyrrole (azapentalene, AZA) was available from previous work in our laboratory [25]. Stock solutions of HSA (1.0 mM) were prepared in 10 mM phosphate-buffered saline (PBS). Stock solutions of AZA and C153 (10.0 mM) were prepared in N,N-dimethylformamide. Ultrapure Milli-Q water was used throughout the experiments.

### 3.2. Preparation of Hypohalous Acids and Haloamines Solutions

Stock solutions of free chlorine (100 mM) were prepared daily by diluting a 12–15% sodium hypochlorite solution in water, and the concentration was determined spectrophotometrically after dilution in 0.01 M NaOH, pH 12 (ε_292 nm_ = 378 M^−1^ cm^−1^) [54]. Hypobromous acid (HOBr) was synthesized by combining 100 mM free chlorine and 200 mM NaBr in water [28]. The stock solutions of N-chlorotaurine (10 mM, Tau-Cl) were prepared immediately before the experiments by adding 10 mM free chlorine to 100 mM taurine in PBS, pH 7.4 [22,55]. The stock solutions of GABA chloramine (GABA-Cl, 10 mM) and the ethyl ester (GABAet-Cl, 10 mM) were prepared immediately before the experiments by adding 10 mM free chlorine to 100 mM GABA or GABA*et* in PBS. The stock solutions of GABA bromamine (GABA-Br) and its ethyl ester (GABAet-Br) were prepared similarly using HOBr.

The stability of the haloamines was assessed by measuring their oxidizing capacity using the 5-thio-2-nitrobenzoic acid (TNB) assay (ε_412nm_ = 14,100 M^−1^ cm^−1^) [22]. TNB was prepared by dissolving 25 mM of DTNB in PBS at pH 7.4 and adjusting the pH to 12 with 0.1 M NaOH. After 10 min, the pH was readjusted to 7.4 with 0.1 M HCl. The resulting TNB solution had a concentration of approximately 2.5 mM and exhibited a 15% decrease in concentration over a week when stored in the refrigerator. The haloamine concentrations were determined by their reaction with TNB, and the change in absorbance at 412 nm after the addition of haloamines was used to calculate their concentration based on the stoichiometry of the reaction:

$$R\text{-}NHCl(Br)\;+\;2\;\;TNB_{\left(412\;nm\right)}⇌R\text{-}NH_{2}+\;DTNB\;+\;Cl^{-}\;or\;Br^{-}$$

### 3.3. Reaction of Haloamines with Molecular Targets: General Procedure

The oxidations of AZA and C153 were monitored by following their intrinsic fluorescence. AZA was excited at 370 nm, and the emission was measured at 430 nm [25]. C153 was excited at 440 nm, and the emission was recorded at 550 nm [51]. Unless stated otherwise, the reaction systems were composed of AZA at 6.25 μM or C153 at 12.5 μM in varying concentrations of haloamines (see figure legends) in PBS, pH 7.4, at 25 °C. The reactions were triggered by the addition of haloamines. When present, the surfactants were added before the addition of the fluorescent probes. The final concentrations of the surfactants were SDS at 40 mM, CTAC at 5.0 mM, and TX-100 at 1.25 mM. These final concentrations are five-fold higher than typical values of their critical micelle concentration [52]. Stock solutions of SDS (400 mM), CTAC (50 mM), and TX-100 (12.5 mM) were prepared in PBS. The stock solutions were diluted in PBS and shaken for 10 min (using an orbital shaker) to achieve the final concentration of surfactants. Then, AZA or C153 was added, and the mixture was shaken for 5 min. Finally, the haloamines were added, and the fluorescence bleaching was monitored by fluorimetry. The reactions were triggered by adding haloamines, and the fluorescence decay was monitored at 360/430 nm (AZA) and 440/550 nm (C153) using a Synergy 2 Multimode microplate reader (BioTek, Winooski, VT, USA). To assess the inhibitory effect of NATA on the oxidation of C153 by haloamines, increasing concentrations of NATA were added to pre-formed SDS micelles (40 mM) along with C153 (12.5 µM). Subsequently, haloamines (200 μM) were introduced to the mixture, and the reaction was incubated for 5 min in PBS at pH 7.4. The inhibition of C153 oxidation was determined by measuring the fluorescence bleaching at 440/550 nm, at which the results were compared in the presence and absence of NATA.

### 3.4. Determination of Second-Order Rate Constants

The reactions were studied by stopped-flow techniques to determine the second-order rate constants. The fast-kinetic experiments were performed using a single-mixing stopped-flow system equipped with a high-intensity LED source and cut-off filters (SX20/LED Stopped-Flow System, Applied Photophysics, Leatherhead, UK). The experiments were performed under pseudo-first-order conditions, where an excess of haloamines (4- to 50-fold) was added to the fluorescent probe (specific values are presented in the figure legends). In a typical experiment, one syringe contained a fluorescent probe, and the second contained an excess of haloamines. The reactions were conducted in PBS at 25 °C. Three determinations were performed for each fluorescent compound or oxidant concentration. The oxidation of AZA was monitored using a 360 nm LED for excitation and a 420 nm long-pass cut-off filter for emission. The oxidation of C153 was monitored using a 435 nm LED for excitation and a 475 nm long-pass cut-off filter for emission. The observed pseudo-first-order constant (k_obs_) was determined by fitting the fluorescence bleaching data to a single exponential decay function (Equation (1)).

(1) $$F=F_{0}\times \;e^{-k_{obs}\times t}$$

The second-order rate constants (k_2_) were obtained assuming a pseudo-first-order condition as follows:

Reaction rate = k_2_ × [A] × [B]

[A] represents the concentration of haloamines, and [B] refers to AZA or C153.

Taken [A] >> [B] → Reaction rate = k_obs_ × [B].

k_obs_ = k_2_ × [A].

Hence, *k*_2_ is the slope of the linear fit of k_obs_ versus [A].

### 3.5. Chemiluminescence Studies

The reactions were monitored by light emission. Unless otherwise stated, the reaction mixtures consisted of 40 mM SDS, 100 μM haloamines, 100 μM H_2_O_2_, and 25 μM luminol in PBS, pH 7.4, at 25 °C. The reactions were conducted in white, flat-bottom microplates with a total volume of 250 μL. The reactions were triggered by adding H_2_O_2_ using the equipment’s automated injector. The reactions were monitored using a plate luminometer (Centro Microplate Luminometer LB960, Berthold Technologies, Oak Ridge, TN, USA). The integrated light emission was used as an analytical parameter for measuring reaction efficiency.

### 3.6. Oxidation of Human Serum Albumin

The oxidation of HSA by haloamines was investigated by monitoring the fluorescence bleaching of the tryptophan residue in the protein. The intrinsic fluorescence of HSA (5.0 μM) was recorded before and 10 min after the addition of haloamines (125 μM), in PBS at pH 7.4 and 25 °C. The excitation wavelength was set to 295 nm, while emission was measured in the range of 310 to 450 nm. To determine the second-order rate constant, stopped-flow experiments were conducted. In these experiments, the reaction medium consisted of HSA (5.0 μM) and haloamines at concentrations ranging from 30 to 250 μM in PBS at pH 7.4 and 25 °C. The experiments were designed as pseudo-first-order reactions by using excess haloamines, with a factor of 6 to 50 times the concentration of HSA. The oxidation process was monitored using a 280 nm LED for excitation, and a 305 nm long-pass cut-off filter for emission.

### 3.7. Bacteria Killing Assay

*Staphylococcus aureus* ATCC 6538 and *Escherichia coli* ATCC 11229, which had been stored in a deep freeze, were cultured on Mueller-Hinton agar. The cells were incubated overnight at 37 °C in tryptic soy broth until they reached a concentration of 1 to 5 × 10^9^ colony-forming units (CFU)/mL. After incubation, the cultures were centrifuged at 1800× *g*, washed twice, resuspended, and then diluted 10-fold in 0.9% NaCl for the tests.

GABA-Cl and GABAet-Cl were tested at concentrations of 10 mM and 50 mM, respectively, and GABA-Br and GABAet-Br at a concentration of 10 µM. GABA and GABAet were used as controls at a concentration of 100 mM. To 3.96 mL of the test or control solution in PBS pre-warmed at 37 °C, 40 µL of the bacterial suspension (*S. aureus* or *E. coli*) in 0.9% NaCl was added at time zero. After 1 to 60 min of incubation at 37 °C, aliquots of 100 µL were mixed with 900 µL of a 1% inactivation solution consisting of 1% methionine and 1% histidine [56]. Aliquots of this suspension were spread onto Mueller-Hinton agar plates in duplicate (50 µL each) using an automatic spiral plater (model WASP 2; Don Whitley, Shipley, UK). The detection limit was 100 CFU/mL, considering both plates and the dilution in the inactivation solution. Plates were grown for 24 h at 37 °C before colonies were counted. Plates with no growth or only a low CFU count were incubated for up to five days to detect bacteria that were attenuated but not killed by the treatment. The results are presented as mean values and standard deviations of three independent experiments. Student’s unpaired *t*-tests were used to test for statistical significance between test and control samples as well as between GABA-Cl and GABAet-Cl and GABA-Br and GABAet-Br, respectively.

## 4. Conclusions

The oxidizing strength of GABA haloamines in hydrophobic microenvironments was significantly increased by esterification. This chemical effect was related to their higher capacity to access the core of the hydrophobic microenvironment. The reactivity of GABAet-Br remained higher compared to GABAet-Cl, consistent with the typical feature of bromamines compared to chloramines. The GABA haloamines can be prepared easily by mixing hypohalous acids in aqueous solutions containing an excess of GABA or GABAet. The stability of the solutions is comparable to that of Tau-Cl, a well-known antiseptic agent. GABAet-Cl and GABAet-Br exhibited more potent activity against *S. aureus* and *E. coli* than the non-esterified GABA-Cl and GABA-Br. In summary, the process of esterification and the resulting increase in hydrophobicity present a new opportunity for the development of more effective haloamines as topical antiseptic agents.

## Data Availability

The original contributions presented in the study are included in the article/Supplementary Materials. Further inquiries can be directed to the corresponding author.

## Supplementary Materials

The following supporting information can be downloaded at: https://www.mdpi.com/article/10.3390/molecules30214227/s1. Figure S1: UV-Vis spectra of haloamines. Figure S2: Comparison of GABA-Cl and GABAet-Cl in the absence of micelles. Figure S3: GABAet-Cl versus GABAet-Br in SDS micelles. Figure S4: Effect of surfactants and the presence of bromide on the oxidation of AZA by GABAet-Cl. Figure S5: Oxidation of C153: GABAet-Br versus GABA-Br in TX-100 micelles.

## Abbreviations

The following abbreviations are used in this manuscript:

AZA	2,5-Bis-(4-methylsulfanyl-phenyl)-1,4-di-p-tolyl-1,4-dihydro-pyrrolo[3,2-b]pyrrole
DTNB	5,5′-dithio-bis-(2-nitrobenzoic acid
C153	Coumarin 153
CMC	Critical micellar concentration
CTAC	Cetyltrimethylammonium chloride
GABA	Gamma-aminobutyric acid
GABA-Cl	Chloramine of GABA
GABA-Br	Bromamine of GABA
GABAet-Cl	Chloramine of the ethyl ester of GABA
GABA-et-Br	Bromamine of the ethyl ester of GABA
HSA	Human serum albumin
NATA	*N*-acetyl-*L*-tryptophanamide
SDS	sodium dodecyl sulfate
Tau-Cl	Chloramine of taurine
Tau-Br	Bromamine of taurine
TX-100	Triton X-100

## References

[1] Weiss S., Klein R., Slivka A., Wei M. (1982). Chlorination of Taurine by Human-Neutrophils—Evidence for Hypochlorous Acid Generation. J. Clin. Investig..

[2] Learn D.B., Fried V.A., Thomas E.L. (1990). Taurine and Hypotaurine Content of Human Leukocytes. J. Leukoc. Biol..

[3] Marquez L.A., Dunford H.B. (1994). Chlorination of Taurine by Myeloperoxidase. Kinetic Evidence for an Enzyme-Bound Intermediate. J. Biol. Chem..

[4] Lin W., Chen H., Chen X., Guo C. (2024). The Roles of Neutrophil-Derived Myeloperoxidase (MPO) in Diseases: The New Progress. Antioxidants.

[5] Jokumsen K.V., Huhle V.H., Hagglund P.M., Davies M.J., Gamon L.F. (2024). Elevated Levels of Iodide Promote Peroxidase-Mediated Protein Iodination and Inhibit Protein Chlorination. Free Radic. Biol. Med..

[6] Frangie C., Daher J. (2022). Role of Myeloperoxidase in Inflammation and Atherosclerosis (Review). Biomed. Rep..

[7] Kim S.O., Shapiro J.P., Cottrill K.A., Collins G.L., Shanthikumar S., Rao P., Ranganathan S., Stick S.M., Orr M.L., Fitzpatrick A.M. (2023). Substrate-Dependent Metabolomic Signatures of Myeloperoxidase Activity in Airway Epithelial Cells: Implications for Early Cystic Fibrosis Lung Disease. Free Radic. Biol. Med..

[8] Nagl M., Arnitz R., Lackner M. (2018). N-Chlorotaurine, a Promising Future Candidate for Topical Therapy of Fungal Infections. Mycopathologia.

[9] Speth C., Rambach G., Windisch A., Neurauter M., Maier H., Nagl M. (2022). Efficacy of Inhaled N-Chlorotaurine in a Mouse Model of Lichtheimia Corymbifera and Aspergillus Fumigatus Pneumonia. J. Fungi.

[10] Walczewska M., Peruń A., Białecka A., Śróttek M., Jamróz W., Dorożyński P., Jachowicz R., Kulinowski P., Nagl M., Gottardi W. (2017). Comparative Analysis of Microbicidal and Anti-Inflammatory Properties of Novel Taurine Bromamine Derivatives and Bromamine T. Adv. Exp. Med. Biol..

[11] Murashevych B., Girenko D., Koshova I., Maslak G., Burmistrov K., Stepanskyi D. (2024). Broad-Purpose Solutions of N-Chlorotaurine: A Convenient Synthetic Approach and Comparative Evaluation of Stability and Antimicrobial Activity. J. Chem..

[12] Arnitz R., Stein M., Bauer P., Lanthaler B., Jamnig H., Scholl-Bürgi S., Stempfl-Al-Jazrawi K., Ulmer H., Baumgartner B., Embacher S. (2018). Tolerability of Inhaled N-Chlorotaurine in Humans: A Double-Blind Randomized Phase I Clinical Study. Ther. Adv. Respir. Dis..

[13] Teuchner B., Nagl M., Schidlbauer A., Ishiko H., Dragosits E., Ulmer H., Aoki K., Ohno S., Mizuki N., Gottardi W. (2005). Tolerability and Efficacy of N-Chlorotaurine in Epidemic Keratoconjunctivitis--a Double-Blind, Randomized, Phase-2 Clinical Trial. J. Ocul. Pharmacol. Ther..

[14] Kyriakopoulos A.M., Nagl M., Gupta R.C., Marcinkiewicz J., Schaffer S.W., El Idrissi A., Murakami S. (2022). Taurine and N-Bromotaurine in Topical Treatment of Psoriasis. Taurine 12: A Conditionally Essential Amino Acid.

[15] Arnhold J., Malle E. (2022). Halogenation Activity of Mammalian Heme Peroxidases. Antioxidants.

[16] Pattison D.I., Davies M.J. (2004). Kinetic Analysis of the Reactions of Hypobromous Acid with Protein Components:  Implications for Cellular Damage and Use of 3-Bromotyrosine as a Marker of Oxidative Stress. Biochemistry.

[17] Ximenes V.F., da Fonseca L.M., de Almeida A.C. (2011). Taurine Bromamine: A Potent Oxidant of Tryptophan Residues in Albumin. Arch. Biochem. Biophys..

[18] Ritter C.L., Malejka-Giganti D. (1989). Oxidations of the Carcinogen N-Hydroxy-N-(2-Fluorenyl)Acetamide by Enzymatically or Chemically Generated Oxidants of Chloride and Bromide. Chem. Res. Toxicol..

[19] De Carvalho Bertozo L., Morgon N.H., De Souza A.R., Ximenes V.F. (2016). Taurine Bromamine: Reactivity of an Endogenous and Exogenous Anti-Inflammatory and Antimicrobial Amino Acid Derivative. Biomolecules.

[20] Baliou S., Sofopoulos M., Goulielmaki M., Spandidos D.A., Ioannou P., Kyriakopoulos A.M., Zoumpourlis V. (2021). Bromamine T, a Stable Active Bromine Compound, Prevents the LPS-induced Inflammatory Response. Int. J. Mol. Med..

[21] Pasich E., Walczewska M., Białecka A., Peruń A., Kasprowicz A., Marcinkiewicz J., Marcinkiewicz J., Schaffer S.W. (2015). Taurine Haloamines and Biofilm: II. Efficacy of Taurine Bromamine and Chlorhexidine Against Selected Microorganisms of Oral Biofilm. Taurine 9.

[22] Marcinkiewicz J. (2010). Taurine Bromamine (TauBr)—Its Role in Immunity and New Perspectives for Clinical Use. J. Biomed. Sci..

[23] Cunningham C., Tipton F.K., Dixon B.F.H. (1998). Conversion of Taurine into N-Chlorotaurine (Taurine Chloramine) and Sulphoacetaldehyde in Response to Oxidative Stress. Biochem. J..

[24] Thomas E.L., Grisham M.B., Jefferson M.M. (1986). Preparation and Characterization of Chloramines. Methods Enzymol..

[25] Pavan N.M., Martins L.M., Augusto L.C., da Silva-Filho L.C., Ximenes V.F. (2022). Development of Fluorescent Azapentalenes to Study the Reactivity of Hypochlorous Acid and Chloramines in Micellar Systems. J. Mol. Liq..

[26] Pilz M., Staats K., Assadian O., Windhager R., Holinka J. (2024). Tolerability of N-Chlorotaurine in Comparison with Routinely Used Antiseptics: An in Vitro Study on Chondrocytes. Pharmacol. Rep..

[27] Peskina A.V., Midwinter R.G., Harwood D.T., Winterbourn C.C. (2005). Chlorine Transfer between Glycine, Taurine, and Histamine: Reaction Rates and Impact on Cellular Reactivity. Free Radic. Biol. Med..

[28] Thomas E.L., Bozeman P.M., Jefferson M.M., King C.C. (1995). Oxidation of Bromide by the Human Leukocyte Enzymes Myeloperoxidase and Eosinophil Peroxidase. Formation of Bromamines. J. Biol. Chem..

[29] Kettle A.J., Albrett A.M., Chapman A.L., Dickerhof N., Forbes L.V., Khalilova I., Turner R. (2014). Measuring Chlorine Bleach in Biology and Medicine. Biochim. Biophys. Acta BBA Gen. Subj..

[30] Ximenes V.F., Padovan C.Z., Carvalho D.A., Fernandes J.R. (2010). Oxidation of Melatonin by Taurine Chloramine. J. Pineal Res..

[31] Marcinkiewicz J., Mak M., Bobek M., Biedroń R., Białecka A., Koprowski M., Kontny E., Maśliński W. (2005). Is There a Role of Taurine Bromamine in Inflammation? Interactive Effects with Nitrite and Hydrogen Peroxide. Inflamm. Res..

[32] Maciążek-Jurczyk M., Sułkowska A. (2015). Spectroscopic Analysis of the Impact of Oxidative Stress on the Structure of Human Serum Albumin (HSA) in Terms of Its Binding Properties. Spectrochim. Acta Part A Mol. Biomol. Spectrosc..

[33] Dypbukt J.M., Bishop C., Brooks W.M., Thong B., Eriksson H., Kettle A.J. (2005). A Sensitive and Selective Assay for Chloramine Production by Myeloperoxidase. Free Radic. Biol. Med..

[34] Carr A.C., Hawkins C.L., Thomas S.R., Stocker R., Frei B. (2001). Relative Reactivities of N-Chloramines and Hypochlorous Acid with Human Plasma Constituents. Free Radic. Biol. Med..

[35] Peskin A.V., Winterbourn C.C. (2001). Kinetics of the Reactions of Hypochlorous Acid and Amino Acid Chloramines with Thiols, Methionine, and Ascorbate. Free Radic. Biol. Med..

[36] Abdeldaim D.T., Mansour F.R. (2018). Micelle-Enhanced Flow Injection Analysis. Rev. Anal. Chem..

[37] Yu Y., Yuan Z., Lu C. (2023). Long-Lasting Chemiluminescence by Aggregation-Induced Emission Surfactant with Ultralow Critical Micelle Concentration. Aggregate.

[38] Skaff O., Pattison D.I., Davies M.J. (2007). Kinetics of Hypobromous Acid-Mediated Oxidation of Lipid Components and Antioxidants. Chem. Res. Toxicol..

[39] Liu Q., Xu X., Fu J., Du Y., Lin L., Bai L., Wang D. (2021). Role of Hypobromous Acid in the Transformation of Polycyclic Aromatic Hydrocarbons during Chlorination. Water Res..

[40] Heeb M.B., Kristiana I., Trogolo D., Arey J.S., von Gunten U. (2017). Formation and Reactivity of Inorganic and Organic Chloramines and Bromamines during Oxidative Water Treatment. Water Res..

[41] Duplâtre G., Ferreira Marques M.F., da Graça Miguel M. (1996). Size of Sodium Dodecyl Sulfate Micelles in Aqueous Solutions as Studied by Positron Annihilation Lifetime Spectroscopy. J. Phys. Chem..

[42] Patel V., Dharaiya N., Ray D., Aswal V.K., Bahadur P. (2014). pH Controlled Size/Shape in CTAB Micelles with Solubilized Polar Additives: A Viscometry, Scattering and Spectral Evaluation. Colloids Surf. A Physicochem. Eng. Asp..

[43] Zhang L., Chai X., Sun P., Yuan B., Jiang B., Zhang X., Liu M. (2019). The Study of the Aggregated Pattern of TX100 Micelle by Using Solvent Paramagnetic Relaxation Enhancements. Molecules.

[44] Kelepouris L., Blanchard G.J. (2003). Dynamics of 7-Azatryptophan and Tryptophan Derivatives in Micellar Media. The Role of Ionic Charge and Substituent Structure. J. Phys. Chem. B.

[45] Fanali G., di Masi A., Trezza V., Marino M., Fasano M., Ascenzi P. (2012). Human Serum Albumin: From Bench to Bedside. Mol. Asp. Med..

[46] Ashraf S., Qaiser H., Tariq S., Khalid A., Makeen H.A., Alhazmi H.A., Ul-Haq Z. (2023). Unraveling the Versatility of Human Serum Albumin—A Comprehensive Review of Its Biological Significance and Therapeutic Potential. Curr. Res. Struct. Biol..

[47] Nishi K., Yamasaki K., Otagiri M. (2020). Serum Albumin, Lipid and Drug Binding. Vertebrate and Invertebrate Respiratory Proteins, Lipoproteins and other Body Fluid Proteins.

[48] de Carvalho Bertozo L., Fernandes A.J.F.C., Yoguim M.I., Bolean M., Ciancaglini P., Ximenes V.F. (2020). Entropy-Driven Binding of Octyl Gallate in Albumin: Failure in the Application of Temperature Effect to Distinguish Dynamic and Static Fluorescence Quenching. J. Mol. Recognit..

[49] Gorudko I.V., Grigorieva D.V., Shamova E.V., Kostevich V.A., Sokolov A.V., Mikhalchik E.V., Cherenkevich S.N., Arnhold J., Panasenko O.M. (2014). Hypohalous Acid-Modified Human Serum Albumin Induces Neutrophil NADPH Oxidase Activation, Degranulation, and Shape Change. Free Radic. Biol. Med..

[50] Vlasova I.I., Sokolov A.V., Kostevich V.A., Mikhalchik E.V., Vasilyev V.B. (2019). Myeloperoxidase-Induced Oxidation of Albumin and Ceruloplasmin: Role of Tyrosines. Biochem. Mosc..

[51] Prazeres T.J.V., Beija M., Fernandes F.V., Marcelino P.G.A., Farinha J.P.S., Martinho J.M.G. (2012). Determination of the Critical Micelle Concentration of Surfactants and Amphiphilic Block Copolymers Using Coumarin 153. Inorganica Chim. Acta.

[52] Karimi M.A., Mozaheb M.A., Hatefi-Mehrjardi A., Tavallali H., Attaran A.M., Shamsi R. (2015). A New Simple Method for Determining the Critical Micelle Concentration of Surfactants Using Surface Plasmon Resonance of Silver Nanoparticles. J. Anal. Sci. Technol..

[53] Marcinkiewicz J., Strus M., Walczewska M., Machul A., Mikołajczyk D. (2013). Influence of Taurine Haloamines auCl and TauBr) on the Development of Pseudomonas Aeruginosa Biofilm: A Preliminary Study. Adv. Exp. Med. Biol..

[54] Kishimoto N. (2019). State of the Art of UV/Chlorine Advanced Oxidation Processes: Their Mechanism, Byproducts Formation, Process Variation, and Applications. J. Water Environ. Technol..

[55] Tokunaga S., Kanayama A., Miyamoto Y. (2007). Modification of IkappaBalpha by Taurine Bromamine Inhibits Tumor Necrosis Factor Alpha-Induced NF-kappaB Activation. Inflamm. Res..

[56] Böttcher B., Sarg B., Lindner H.H., Nagl M. (2017). Inactivation of Microbicidal Active Halogen Compounds by Sodium Thiosulphate and Histidine/Methionine for Time-Kill Assays. J. Microbiol. Methods.

