# Supplementary material for: Haloamines of the Neurotransmitter γ-Aminobutyric Acid (GABA) and Its Ethyl Ester: Mild Oxidants for Reactions in Hydrophobic Microenvironments and Bactericidal Activity
Source: Molecules. 2025 Oct 29;30(21):4227. doi: 10.3390/molecules30214227 (PMC12610792; doi:10.3390/molecules30214227)
Supplement: Supplementary file 1 [file molecules-30-04227-s001.zip › molecules-3935387-supplementary.pdf]

Supplementary Information

Haloamines of the Neurotransmitter γ-Aminobutyric Acid (GABA) and Its Ethyl Ester: Mild Oxidants for Reactions in Hydrophobic Microenvironments and Bactericidal Activity

Luiza de Carvalho Bertozo [1], Markus Nagl [2] and Valdecir Farias Ximenes [1,*]

[1] São Paulo State University (UNESP), School of Sciences, Bauru 17033-360, Brazil

luiza.bertozo@unesp.br

[2] Institute of Hygiene and Medical Microbiology, Medical University of Innsbruck, 6020 Innsbruck, Austria

Email: m.nagl@i-med.ac.at

*Correspondence: Email: valdecir.ximenes@unesp.br

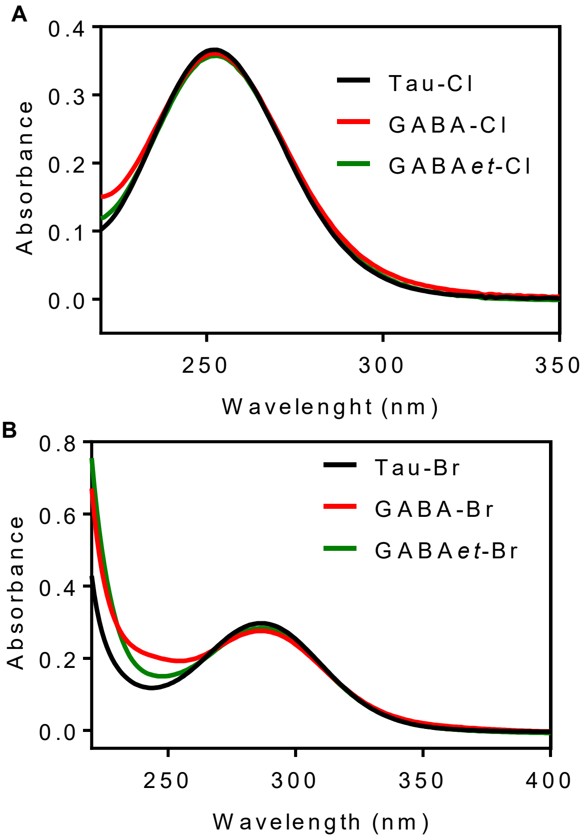

Figure S1. (**A**) UV absorption spectra of taurine chloramine (Tau-Cl), GABA chloramine (GABA-Cl), and ethyl ester of GABA chloramine (GABA*et*-Cl). (**B**) UV absorption spectra of taurine bromamine (Tau-Br), GABA bromamine (GABA-Cl), and ethyl ester of GABA bromamine. Experimental conditions: taurine, GABA and GABA*et* 10 mM, HOCl 1.0 mM, HOBr 1.0 mM in 50 mM phosphate buffer, pH 9.0.

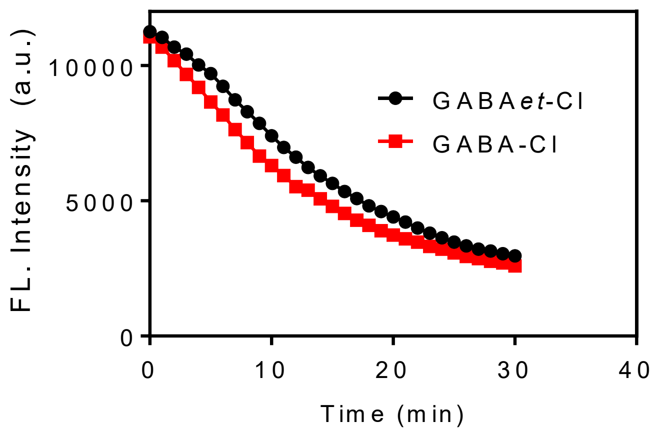

Figure S2. Oxidation of AZA in the absence of SDS. GABA-Cl versus GABA*et*-Cl. Time-dependent emission decay. Final concentrations: AZA 10 μM, GABA-Cl or GABA*et*-Cl 200 μM in PBS:ethanol (1:1), pH 7.4, 25°C.

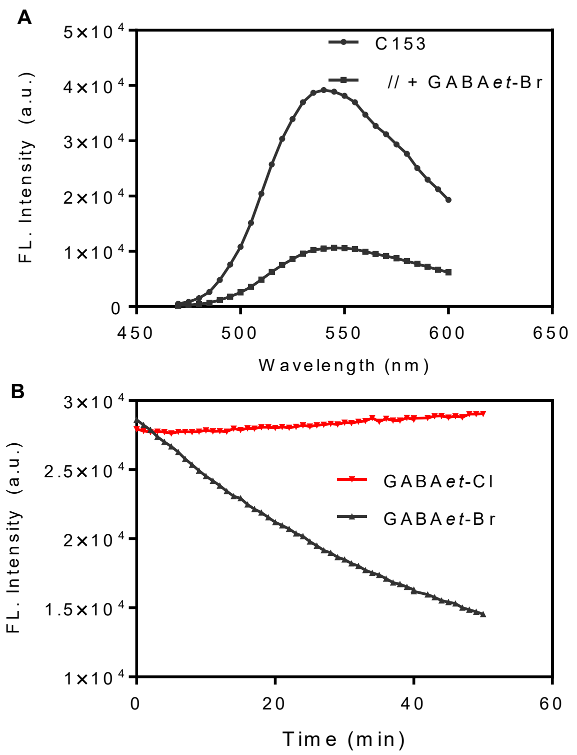

Figure. S3. Oxidation of C153: GABA*et*-Cl versus GABA*et*-Br in SDS micelles. **A**) C153 emission spectrum before and after the addition of GABA*et*-Br. (**B**) Time-dependent emission decay (440/550 nm). Final concentrations: SDS 40 mM in PBS pH 7.4, C153 10 μM, GABA-Cl or GABA*et*-Cl 400 μM.

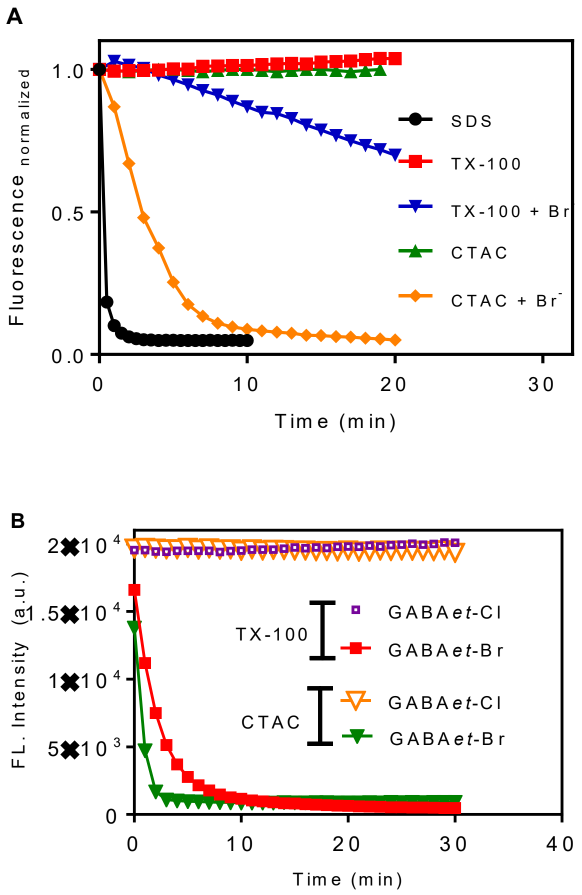

Figure S4. (**A**) Effect of surfactants and the presence of bromide on the oxidation of AZA by GABA*et*-Cl. Final concentrations: SDS 40 mM, TX-100 5 mM, CTAC 10 mM, Br^- 10 mM, AZA 10 µM, GABA*et*-Cl 1.0 mM, PBS pH 7.4, 25°C. (**B**) Comparison between GABA*et*-Cl and GABA*et*-Br. Final concentrations: SDS 40 mM, TX-100 5 mM, CTAC 10 mM, AZA 10 µM, GABA*et*-Cl 1.0 mM, GABA*et*-Br 1.0 mM PBS pH 7.4, 25°C.

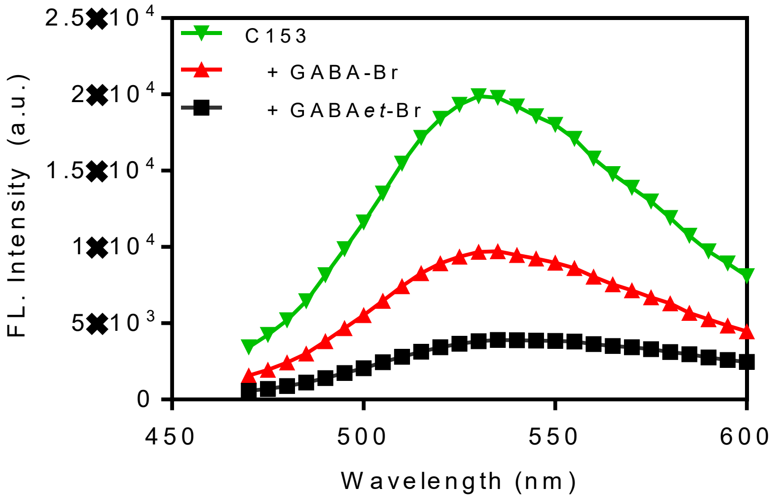

Figure S5. Oxidation of C153: GABA*et*-Br versus GABA-Br in TX-100 micelles. Final concentrations: TX-100 5 mM in PBS pH 7.4, C153 10 μM, GABA-Br and GABA*et*-Br 250 μM.
